# Function and mechanism of action of the small regulatory RNA ArcZ in *Enterobacterales*

**DOI:** 10.1261/rna.080010.124

**Published:** 2024-09

**Authors:** Quentin Dubois, Typhaine Brual, Charlotte Oriol, Pierre Mandin, Guy Condemine, Erwan Gueguen

**Affiliations:** 1 Université Lyon, Université Claude Bernard Lyon 1, CNRS, INSA Lyon, UMR5240 MAP Lyon, France; 2 CNRS, Aix-Marseille Université, Laboratoire de Chimie Bactérienne, UMR7283, IMM, IM2B, F-13009 Marseille, France

**Keywords:** ArcZ, sRNA, posttranscriptional regulation, virulence, stress response, RNase E

## Abstract

ArcZ is a small regulatory RNA conserved in *Enterobacterales*. It is an Hfq-dependent RNA that is cleaved by RNase E in a processed form of 55–60 nucleotides. This processed form is highly conserved for controlling the expression of target mRNAs. ArcZ expression is induced by abundant oxygen levels and reaches its peak during the stationary growth phase. This control is mediated by the oxygen-responsive two-component system ArcAB, leading to the repression of *arcZ* transcription under low-oxygen conditions in most bacteria in which it has been studied. ArcZ displays multiple targets, and it can control up to 10% of a genome and interact directly with more than 300 mRNAs in *Escherichia coli* and *Salmonella enterica*. ArcZ displays a multifaceted ability to regulate its targets through diverse mechanisms such as RNase recruitment, modulation of ribosome accessibility on the mRNA, and interaction with translational enhancing regions. By influencing stress response, motility, and virulence through the regulation of master regulators such as FlhDC or RpoS, ArcZ emerges as a major orchestrator of cell physiology within *Enterobacterales*.

## INTRODUCTION

In bacteria, adaptation to environmental changes, production of secondary metabolites, or virulence are highly controlled and regulated processes that allow the genes involved in these pathways to be expressed at the right time (Schneider et al. 2009). Such control can occur directly on the DNA via transcriptional regulation, on the mRNA via posttranscriptional regulation, or on the proteins themselves via posttranslational regulation. Major classes of posttranscriptional regulators are riboswitches and antisense RNAs (asRNAs) that act in *cis* or small noncoding RNAs that act in *trans* (sRNAs). sRNAs are the most abundant class of posttranscriptional regulators (for review, see Eichner et al. 2022).

asRNAs are transcribed in the reverse sense of the target gene. They pair with the mRNA, and this pairing leads to the degradation of both RNAs (Dadzie et al. 2013). On the other hand, sRNAs are small untranslated RNAs, generally short, that regulate their mRNA targets posttranscriptionally (for review, see Eichner et al. 2022). These sRNAs base-pair directly to target mRNAs, often with the help of a chaperone-like Hfq or ProQ. In most cases, these sRNAs pair to the 5′-untranslated region (UTR) region of the target mRNA. In this way, sRNAs can have a repressive effect on mRNA expression, either by hybridizing to the ribosome-binding site (RBS) and/or by recruiting an RNase to degrade the mRNA. They can also have an activating effect on mRNA expression by stabilizing and protecting mRNAs, or by activating translation initiation, for instance by preventing the formation of a translation-inhibiting structure (for review, see Dutta and Srivastava 2018).

The study of sRNA function has taken a major turn since the advent of new high-throughput sequencing methods. These new massive cDNA sequencing techniques have led to the invention of global methods, RIP-seq, CLIP-seq, CLASH, RIL-seq, GRIL-seq, MAPS, and Term-seq, which enable the study of the full range of interactions between Hfq, sRNAs, and their target mRNAs that occur in vivo (for review, see Saliba et al. 2017).

**FIGURE 1.**
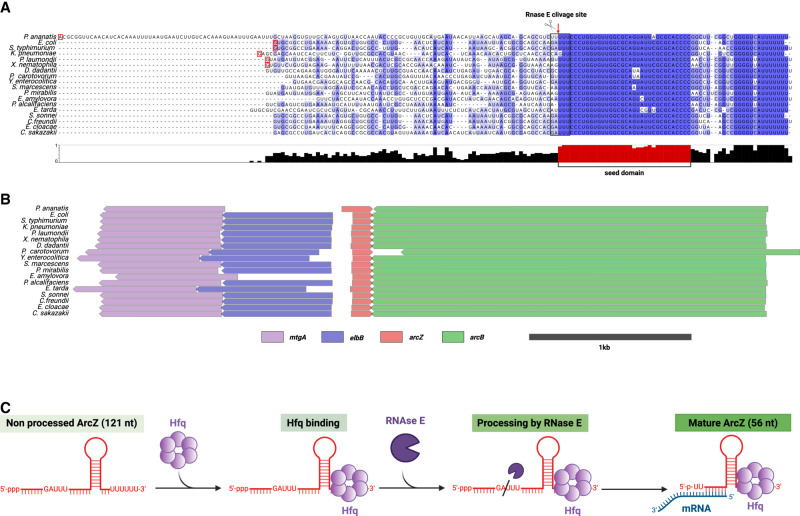
ArcZ, a cleaved sRNA that is highly conserved in its 3′ part. (*A*) Alignment of ArcZ sequence from *Pantoea ananatis* (NZ_CM012203), *Escherichia coli* (NC_000913.3), *Salmonella typhimurium* (NC_003197.2), *Klebsiella pneumoniae* (NC_009648), *Photorhabdus laumondii* (CP024901.1), *Xenorhabdus nematophila* (CP060401.1), *Dickeya dadantii* (CP002038.1), *Pectobacterium carotovorum* (CP051652.1), *Yersinia enterocolitica* (CP107102.1), *Serratia marcescens* (CP139958.1), *Proteus mirabilis* (CP045257.1), *Erwinia amylovora* (FN666575.1), *Providencia alcalifaciens* (CP084296.1), *Edwardsiella tarda* (CP084506.1), *Shigella sonnei* (CP026802.1), *Citrobacter freundii* (CP049015.1), *Enterobacter cloacae* (CP001918.1), and *Cronobacter sakazakii* (CP011047.1). Conservation score is plotted *below*, and the conserved region is colored in red. This alignment was carried out using ClustalW and Jalview (Thompson et al. 2003; Clamp et al. 2004; Troshin et al. 2011). The red squares correspond to known transcription starts. (*B*) Synteny analysis of chromosomal regions surrounding ArcZ was performed using AnnoView (Wei et al. 2024). The same genome accession numbers as *A* were used. (*C*) Model of ArcZ maturation in *E. coli.* The stem–loop represents the Rho-independent transcriptional termination site of *arcZ*. The 3′ region of ArcZ is recognized by Hfq through binding. RNase E cleaves ArcZ at a consensus sequence, producing a mature processed form of ArcZ that binds to target mRNA.

ArcZ is a small RNA found in most *Enterobacterales* bacteria (Fig. 1A) and its role has been more specifically studied in bacteria such as *Salmonella typhimurium*, *Dickeya dadantii*, *Escherichia coli*, *Erwinia amylovora*, and *Photorhabdus* sp. ArcZ was identified in initial genome-wide sRNA screenings of *E. coli* and named either SraH (Argaman et al. 2001) or RyhA (Wassarman et al. 2001). The sequence of this small RNA is relatively well conserved among the different species in which it occurs, especially at the 3′ end (Fig. 1A).

The transcriptional start site (TSS) of ArcZ has been identified precisely in *Pantoea ananatis*, *E. coli* K12, *S. typhimurium*, *Klebsiella pneumoniae*, *Photorhabdus laumondii*, and *Xenorhabdus nematophila* by 5′RACE or genome-wide transcription start site profiling (Fig. 1A, +1 indicated with a red frame) (Argaman et al. 2001; Kim et al. 2012; Kröger et al. 2012; Shin et al. 2019; Neubacher et al. 2020). It usually ranges in size from 121 to 130 nt, except in *P. ananatis*, where the 5′ end is particularly long (Soper et al. 2010; Shin et al. 2019; Neubacher et al. 2020). On the chromosome, the gene encoding this sRNA is located downstream from the *arcB* gene, in the reverse direction (Fig. 1B). The 3′ end of *arcZ* usually overlaps the 3′ end of the *arcB* gene by ten bases and ends at a Rho-independent terminator (Fig. 1B; Argaman et al. 2001). *arcZ* does not overlap *arcB* in *Pectobacterium carotovorum* (Wang et al. 2018). Due to its genomic location, the *sraH*/*ryhA* gene has been renamed as *arcZ* (referring to arc-associated sRNA Z) (Papenfort et al. 2009). This small regulatory RNA targets key regulators (*rpoS*, *flhD*, *lrp*) involved in various functions in the bacterial cell, playing a pleiotropic role in diverse bacterial species (Papenfort et al. 2009). For instance, a transcriptome analysis carried out in *E. amylovora* revealed that ArcZ regulates the expression of 10% of its genome (Schachterle et al. 2019a). Recent RIL-seq data have shown that ArcZ may interact directly with more than 10% of mRNAs in the *E. coli* and *Salmonella* genomes. ArcZ may have one of the largest target regulons for sRNA (Melamed et al. 2016; Liu et al. 2023). However, the *arcZ* gene is not essential in any of the bacteria where it has been studied, and mutated strains lacking this gene survive in laboratory conditions. The purpose of this review is to compile all presently accessible data on ArcZ in various bacterial species of the *Enterobacterales* order. We will discuss the identified targets of ArcZ and the molecular process through which ArcZ can regulate their expression.

## WHEN AND HOW IS A MATURE FORM OF ArcZ OBTAINED?

### *arcZ* is expressed during the stationary phase and in aerobic conditions

The sRNA ArcZ is regulated by the two-component system ArcAB. ArcB is a sensor kinase that can transfer its phosphate to ArcA under anoxic conditions, thereby activating this transcriptional regulator (Brown et al. 2022). Once activated, phosphorylated ArcA represses *arcZ* transcription by binding to the *arcZ* promoter region. Additionally, *arcB* mRNA directly contributes to the repression of ArcZ. It is speculated that *arcB* mRNA acts as an asRNA and destabilizes ArcZ through pairing with it. Therefore, the ArcAB system represses the transcription of ArcZ in situations of limited oxygen supply. ArcZ is expressed under high oxygen conditions (Mandin and Gottesman, 2010). Additionally, ArcZ exhibits maximum levels during the stationary growth phase (Chen and Gottesman 2017). ArcZ also directly represses *arcB* transcription in *E. coli*, providing a negative feedback loop that may affect the function of the ArcA–ArcB regulon (Mandin and Gottesman 2010).

After transcription, ArcZ is recognized by the chaperone Hfq and is rapidly cleaved by RNase E into a shorter 56 nt form (Fig. 1A,C). This processed form corresponds to the 3′ part of ArcZ, highly conserved in *Enterobacterales*, which is the active form that can enhance or repress the expression of its target mRNAs (Fig. 1A,C; Chao et al. 2017).

### Hfq and RNase E, two key players in ArcZ functions

Hfq binds broadly to mRNAs, sRNAs, and ribosomal RNAs (for review, see Updegrove et al. 2016). It is the first protein chaperone discovered to bind with sRNAs (for review, see Vogel and Luisi 2011). It is a hexameric protein abundant in many bacteria and crucial for the stability of mRNA and sRNA expression. As an RNA chaperone, Hfq binds to UA-rich sequences of sRNAs and promotes hybridization of sRNAs to their target mRNAs, which causes either negative or positive regulation of gene expression. Thus, Hfq could be considered as a catalyst that stabilizes the sRNA and promotes the meeting and binding between the sRNA and the mRNA it regulates. It is also possible that Hfq assists sRNA–mRNA binding by modifying the structure of the mRNA upon its own binding (Geissmann and Touati 2004). Like many other sRNAs, ArcZ relies on Hfq for binding to target mRNAs (Fig. 1C; Soper et al. 2010). Hfq can increase the rate of binding of ArcZ to its mRNA targets and the stability of the ArcZ*/*mRNA complex (Soper et al. 2010).

Hfq possesses two faces—a proximal face that enables it to bind to sRNAs and a distal face that facilitates its binding to mRNAs. The consensus binding site for Hfq at the mRNA level corresponds to the motif (AAN)_4_. This repeat is commonly located in the 5′ UTR of target mRNAs, as evidenced by studies carried out on *rpoS* or *mutS* in *E. coli*. It has been proposed that the binding of Hfq alone to *mutS* mRNA creates a structure that inhibits translation, while ArcZ bound to Hfq binds to a sequence near the RBS of *mutS*, preventing ribosome binding (Soper et al. 2010; Chen and Gottesman 2017). Another known function of Hfq is to protect mRNAs and sRNAs from degradation by RNase E. In the case of DsrA and RyhB sRNAs, Hfq binds to these sRNAs at the RNase E cleavage site, preventing their degradation (Moll et al. 2003).

RNase E also plays a crucial role in the maturation of specific sRNAs, including MicL and ArcZ, by processing them into functional forms (Guo et al. 2014; Chao et al. 2017). These sRNAs undergo cleavage by RNase E, resulting in a shorter and stable form that corresponds to the 3′ end (Updegrove et al. 2019). In vivo*,* two forms of *E. coli* ArcZ have been identified: a full 121 nt form and a processed 56 nt form, which corresponds to the 3′ end of ArcZ. Only the short form of ArcZ has the ability to interact with its mRNA targets, resulting in the activation or repression of their translation and/or stability (Fig. 1C; Chao et al. 2017). The short form was also detected by northern blotting in *S. typhimurium*, *D. dadantii*, *Photorhabdus*, and *Xenorhabdus* sp. (Papenfort et al. 2009; Yuan et al. 2019; Neubacher et al. 2020). The RNase E cleavage site of *E. coli* ArcZ consists of a minimum consensus sequence of 5 nt: R(G/A)N(G/A/U/C)W(A/U)UU with the cut occurring between the nucleotide sequences RN and WUU (Fig. 1C). Hfq is required to obtain a unique cleavage. In its absence, RNase E cleaves the ArcZ transcript into various fragments, failing to generate the functional 56 nt-long ArcZ (Chao et al. 2017). Another important factor for the unique cleavage of the full-length form of ArcZ by RNase E at the consensus site is the presence of the highly conserved sequence CCCUGGUGUUGGCGCA immediately following the consensus cleavage site (Chao et al. 2017). Indeed, ArcZ has other potential consensus sites for cleavage by the RNase E, but only one site is cleaved to yield the transcript of 56 nt in *E. coli* (Fig. 1).

The most likely hypothesis to explain why RNase E cleavage of ArcZ is essential to produce a functional sRNA is that the 5′ region of ArcZ prevents the conserved 3′ sequence from being free for base-pairing with mRNAs. Cleavage releases the 3′ part of ArcZ, enabling its base-pairing with its mRNA targets. Therefore, the chaperone Hfq and RNase E are two essential players in the processing of ArcZ into a shorter sRNA of 56 nt, but also for the stabilization and pairing of this sRNA with its targets (Chao et al. 2017).

## THE mRNA TARGETS OF ArcZ IN ENTEROBACTERALES

### ArcZ regulates the general stress response

**FIGURE 2.**
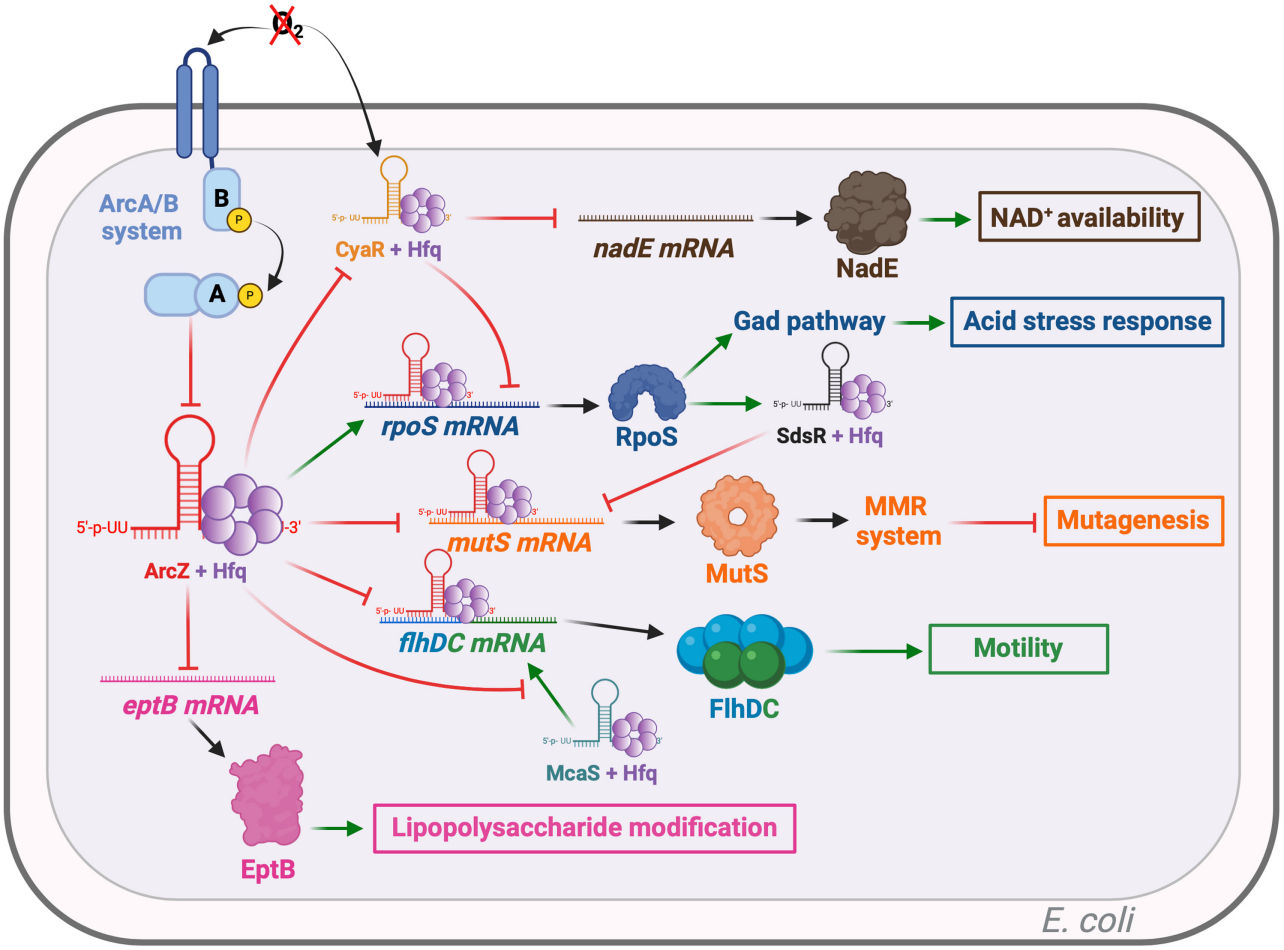
ArcZ targets in *Escherichia coli*. Green arrows indicate activation, red arrows indicate repression, and black arrows indicate either translation or activity. CyaR is an sRNA capable of repressing the translation of *nadE* mRNA, which encodes an enzyme involved in NAD^+^ biosynthesis but is also capable of repressing the translation of *rpoS* mRNA. ArcZ degrades CyaR via RNase E, thereby increasing the translation of *nadE* and the availability of NAD^+^ and *rpoS.* ArcZ enhances the translation of *rpoS* mRNA, which induces the Gad pathway, leading to better acid stress resistance. Additionally, ArcZ reduces the translation of *mutS* mRNA, both directly and indirectly, by activating the translation of *rpoS*, which in turn transcribes SdsR. SdsR directly represses *mutS* translation. ArcZ also directly represses *flhDC* translation and competes with the McaS sRNA, which has a common *flhDC* mRNA pairing site with ArcZ. McaS activates *flhDC* translation, while ArcZ inhibits LPS modification by repressing *eptB* mRNA translation. Figure was created with BioRender.com.

The RpoS-mediated general stress response in *E. coli* has been reviewed extensively (Battesti et al. 2011). The first well-defined target of ArcZ in *E. coli* is the *rpoS* mRNA, as two studies pinpointed this regulation and detailed its molecular mechanism (Fig. 2; Mandin and Gottesman 2010; Soper et al. 2010). RpoS (σ^38^) is an alternative sigma factor responsible for activating genes that enhance resistance against various stresses, including the *gad* genes during acid stress and the *ots* genes during cold stress, through its interaction with RNA polymerase. Directly or not, RpoS regulates ∼500 genes in *E. coli*. During the exponential phase of growth, RpoS production is repressed at various levels, while it is activated during the stationary phase. At the posttranscriptional level, the regulation of *rpoS* is complex: the sRNAs ArcZ, DsrA, and RprA are involved in activating the translation of the *rpoS* mRNA, while the sRNAs OxyS and CyaR repress it (Mandin and Gottesman 2010). In addition to the aforementioned regulation of *arcZ*, the transcription of these various sRNAs is itself regulated. *dsrA* expression is triggered during low-temperature conditions, *rprA* expression is induced through the Rcs phosphorelay, *oxyS* is induced by H_2_O_2_ oxidative stress through the OxyR regulator, and *cyaR* is regulated by CRP (Colland et al. 2000). These sRNAs, except DsrA, are known to be strictly dependent on the chaperone Hfq, without which they can no longer regulate their target (Soper et al. 2010).

During acid stress in *E. coli*, ArcZ, by promoting the translation of *rpoS* mRNA, increases the synthesis of GadX, which in turn activates the transcription of the *gadE* gene. GadE is the primary transcriptional activator of the *gadA* and *gadB* genes, which encode glutamate decarboxylases, as well as *gadD*, which encodes a glutamate transporter (Fig. 2; Castanié-Cornet et al. 2010; Bak et al. 2014). The harmful impact of acid stress is thus counteracted by the production of GABA via the decarboxylation of glutamic acid (De Biase et al. 1999).

Another aspect of ArcZ's influence on *rpoS* mRNA operates through regulatory cascades, specifically through the interaction between ArcZ and CyaR (Fig. 2). CyaR transcription is controlled by the global transcriptional repressor CRP, the CpxAR two-component system, and the sigma factor RpoE (De Lay and Gottesman 2012). CyaR has the ability to interact with *rpoS* mRNA and to downregulate *rpoS* mRNA expression (Fig. 2; Kim and Lee 2020). ArcZ has the ability to directly interact with CyaR and inhibit its function by causing its degradation by the RNase E (Kim and Lee 2020). Conversely, CyaR has no effect on the activity of ArcZ (Iosub et al. 2020). Moreover, since the interaction region between ArcZ and CyaR corresponds to their *rpoS* mRNA binding sites, there may be competition between these two sRNAs for interacting with the *rpoS* mRNA (Kim and Lee 2020). CyaR also represses the translation of *nadE*, which encodes an enzyme involved in NAD^+^ biosynthesis (Hughes et al. 1988). Under anaerobic conditions, ArcZ, which is absent, is unable to repress the action of CyaR. Thus, CyaR reduces the concentration of NAD^+^ by repressing *nadE* translation. Conversely, in aerobiosis, ArcZ prevents CyaR action, providing greater NAD^+^ availability (Fig. 2). These ArcZ–CyaR interactions could thus enhance the regulation of *rpoS* expression, allowing *E. coli* to respond more effectively to various stresses (Kim and Lee 2020).

In *E. coli*, ArcZ directly represses *mutS* mRNA translation (Fig. 2). Bacteria under stress accumulate mutations to better survive and adapt (for review, see Foster 2007). The DNA mismatch repair (MMR) system limits the occurrence of mutations (Wyrzykowski and Volkert 2003). The MutS protein, a crucial component of the MMR system, identifies mispaired bases in DNA and triggers the repair process via the MMR system (Su and Modrich 1986). The repression of *mutS* by ArcZ occurs during the stationary growth phase when ArcZ is abundant. Due to this repression, the MMR system is no longer active and an increase in unrepaired mutations is observed (Chen and Gottesman 2017). Furthermore, the sRNA SdsR directly represses *mutS* (Chen and Gottesman 2017). SdsR is transcribed by the RNA polymerase only when this latter is associated with RpoS. Since ArcZ is able to activate the translation of *rpoS* mRNA in *E. coli*, ArcZ represses *mutS* both directly by acting as a posttranscriptional repressor on *mutS* mRNA and indirectly through SdsR. The repression of *mutS* by ArcZ contributes to stress-induced mutagenesis in *E. coli* (Fig. 2).

**FIGURE 3.**
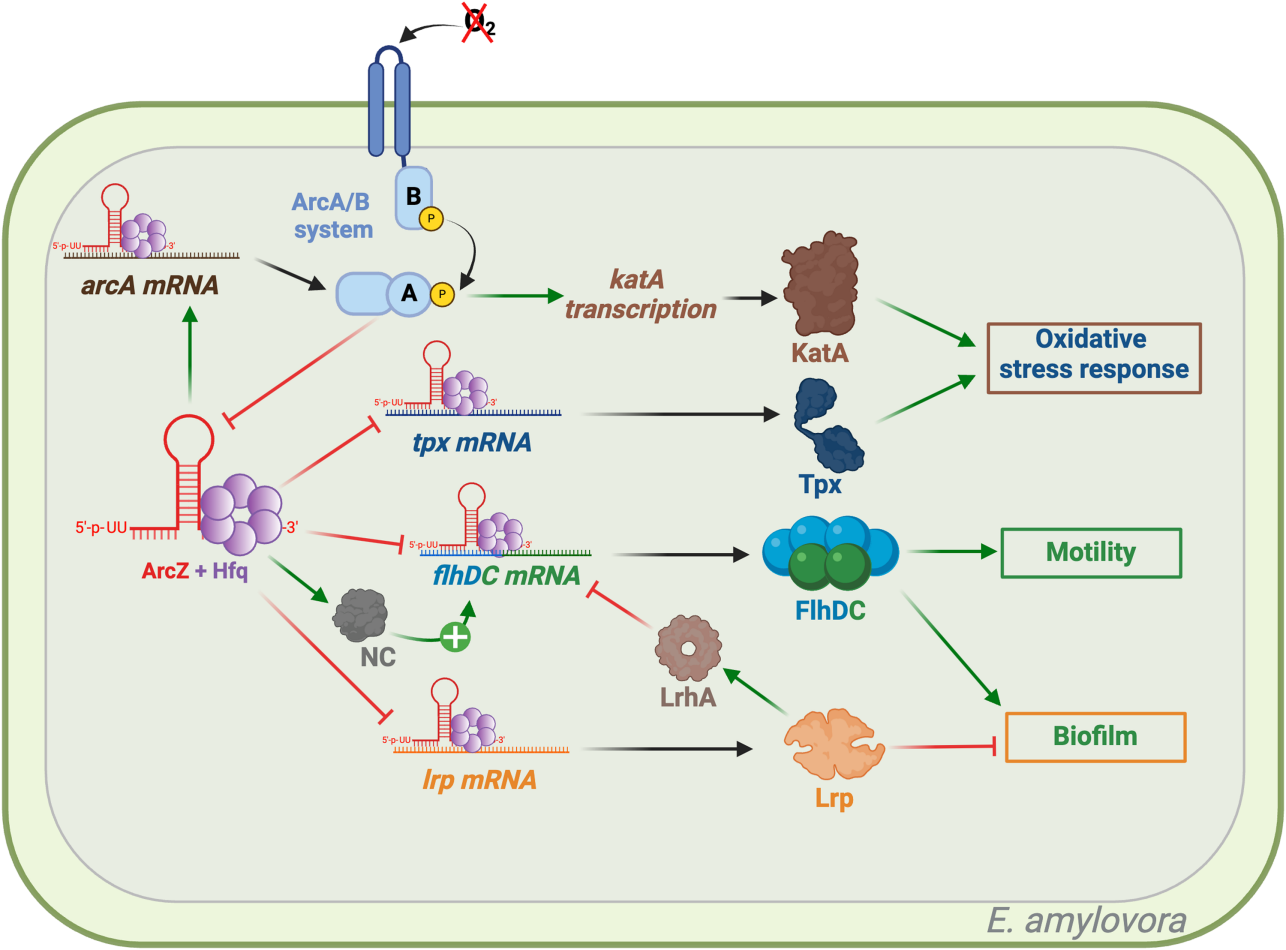
ArcZ targets in *Erwinia amylovora*. Green arrows indicate activation, red arrows indicate repression, and black arrows indicate either translation or activity. ArcZ plays a role in regulating the response to oxidative stress by modulating the levels of the antioxidant enzymes KatA and Tpx. The abbreviation NC stands for “Noncharacterized” protein. ArcZ modulates motility and biofilm formation through the control of *flhDC* and *lrp* translation. Figure was created with BioRender.com.

*Erwinia amylovora* is a phytopathogenic bacterium that causes fire blight and has a wide range of host species within the *Rosaceae* family (e.g., apple, pear, and raspberry) (for review, see Piqué et al. 2015). ArcZ is required for the full virulence of *E. amylovora* (Zeng et al. 2013). In *E. amylovora*, ArcZ modulates the levels of the catalase KatA and of the thiol peroxidase Tpx (Fig. 3). These two enzymes are essential for *E. amylovora* to fight against the free radicals produced by the plant during infection such as hydrogen peroxide produced during apple infection (Santander et al. 2018). ArcZ indirectly regulates *katA* at the transcriptional level through the ArcA regulator. By increasing the translation of ArcA, which is a transcriptional activator of *katA*, ArcZ enhances the response to oxidative stress (Fig. 3; Schachterle et al. 2019a). Previously, it was demonstrated that the ArcAB system in *E. coli* represses the expression of *arcZ* (Fig. 3). If such a regulation exists in *E. amylovora*, the three-way interaction of ArcZ–ArcA–KatA should create a positive feedback loop, increasing the amount of KatA. Conversely, ArcZ directly controls *tpx* at the posttranscriptional level by binding to its mRNA, resulting in a reduced level of the thiol peroxidase Tpx (Fig. 3). Thus, through these two regulatory modes, ArcZ finely tunes the cellular response to oxidative stress based on oxygen availability and oxidative status (Schachterle et al. 2019a). Posttranscriptional repression of *tpx* was also described in *S. typhimurium* (Papenfort et al. 2009).

### ArcZ targets mRNAs involved in virulence

ArcZ is also able to regulate genes important for bacterial virulence, such as motility, biofilm formation, and secretion of antimicrobial compounds.

**FIGURE 4.**
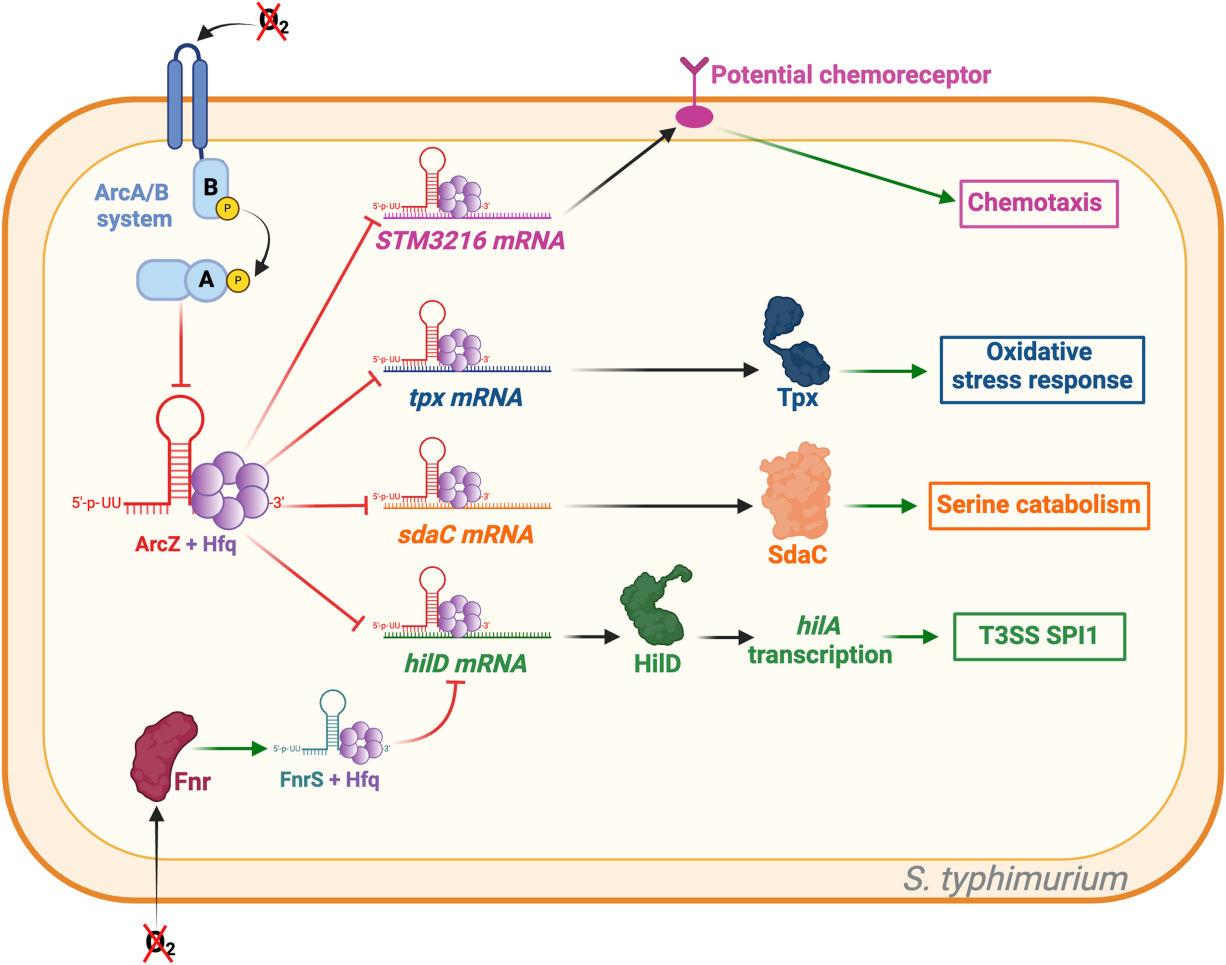
ArcZ targets in *Salmonella typhimurium*. Green arrows indicate activation, red arrows indicate repression, and black arrows indicate either translation or activity. ArcZ represses the expression of STM3216, a potential chemoreceptor acquired through horizontal gene transfer. As previously described, ArcZ modulates the response to oxidative stress by repressing *tpx* mRNA. Additionally, ArcZ represses the translation of *sdaC* mRNA, which is involved in serine catabolism. ArcZ and FnrS inhibit the translation of *hilD*, which is responsible for activating the T3SS synthesis. However, *fnrS* is transcribed under anoxic conditions due to Fnr, whereas *arcZ* is transcribed under strong aerobic conditions. Therefore, the activation of T3SS is limited by the presence of oxygen. Figure was created with BioRender.com.

*Salmonella typhimurium* is a bacterium that is commonly associated with food poisoning. The bacterium's ability to cause disease is primarily attributed to the secretion of virulence factors through a type III secretion system encoded by a pathogenicity island called SPI1. This T3SS enables the secretion of effectors that facilitate the internalization of bacterial cells into host cells. The *hilA* gene is crucial for the synthesis of the *S. typhimurium* T3SS as it activates the T3SS structural genes present in SPI1. *hilA* transcription is regulated by HilC, HilD, and RtsA (Ellermeier et al. 2005). The sRNAs ArcZ and FnrS indirectly repress *hilA* expression by repressing the translation of *hilD* mRNA through direct interaction, but this process relies on the oxygen levels and can be antagonistic. In aerobic conditions, ArcZ is expressed and represses the translation of *hilD*. On the other hand, the two-component system Fnr activates the transcription of FnrS in anoxia, which in turn represses the translation of *hilD* (Fig. 4; Durand and Storz 2010; Kim et al. 2019). This regulatory network enables the most efficient expression of T3SS genes when exposed to fluctuating oxygen levels (Kim et al. 2019).

Interestingly, ArcZ was the first sRNA found to bind to the mRNA of a horizontally acquired virulence gene. This gene, known as STM3216, is specific to *S. typhimurium*, and it is predicted to function as a receptor involved in chemotaxis. ArcZ directly inhibits the translation of STM3216 mRNA (Fig. 4). This discovery highlights the potential of sRNAs to regulate the expression of horizontally acquired genes (Papenfort et al. 2009). ArcZ also controls biofilm formation in *S. typhimurium*, through the transcriptional regulator CsgD. CsgD regulates several genes responsible for curli's assembly, transport, and structural component synthesis. These components are important for biofilm formation (Hammar et al. 1995; Mika and Hengge 2014). CsgD is also the main regulator of the expression of the rdar morphotype, which relates to multicellular behavior characterized by the production of adhesive extracellular matrix and curli expression (for review, see Römling 2005). CsgD is regulated by RpoS, but ArcZ has been shown to partially regulate *csgD* independently of RpoS (Monteiro et al. 2012). Furthermore, ArcZ appears to regulate attachment to surfaces for biofilm formation by repressing the synthesis of type 1 fimbriae (Monteiro et al. 2012).

Escape from the host immune system is also a crucial aspect for successful pathogen infection. The bacterial surface lipopolysaccharides (LPS) are perceived by the host immune system as a foreign element. Consequently, bacteria synthesize enzymes that modify LPS to evade recognition. In *E. coli*, *eptB* encodes an LPS-modifying enzyme. The synthesis of EptB is under the control of the sigma factor RpoE. *eptB* mRNA is directly repressed by ArcZ in *E. coli* (Fig. 2; Moon et al. 2013).

Motility is an essential virulence factor for flagellated bacteria such as *E. coli*, *E. amylovora*, *D. dadantii*, and many others. In *E. coli*, the major regulator of motility is FlhDC, a class I transcriptional activator of the flagellar regulatory cascade (Zhao et al. 2007). In *E. coli*, ArcZ binds directly to *flhD* mRNA and inhibits its translation, along with that of *flhC*, since they are included in the same operon. Consequently, the motility of *E. coli* is reduced when ArcZ is expressed (Fig. 2; De Lay and Gottesman 2012).

A mechanistically identical repression of *flhDC* by ArcZ is also observed in *E. amylovora* (Fig. 3). Interestingly, in contrast to results in *E. coli*, this posttranscriptional repression of *flhDC* by ArcZ actually enhances the motility of *E. amylovora*. This difference could be explained by an indirect activation of *flhDC* by ArcZ in *E. amylovora*, not present in *E. coli* (Schachterle and Sundin 2019; Schachterle et al. 2019b). LrhA is a direct transcriptional repressor of *flhDC*. In *E. coli*, the leucine-responsive regulatory protein (Lrp) binds to the *lrhA* promoter, which leads to its transcriptional activation (Kroner et al. 2019). In *E. amylovora*, ArcZ directly interacts with *lrp* mRNA to posttranscriptionally repress it. Thus, in *E. amylovora*, ArcZ directly represses *flhDC* while indirectly promoting *flhDC* transcription through the *lrp–lrhA* pair. Therefore, the ArcZ–Lrp–FlhDC trio operates as a “feed-forward” type of regulatory loop (Schachterle and Sundin 2019). This regulatory loop may accelerate the response through faster FlhDC production when ArcZ levels vary. This effect has been previously demonstrated for the “feed-forward” regulatory loop Fur–SodA–RyhB (Semsey 2014). It is worth noting that FlhDC activates levan production and that Lrp represses the production of the exopolysaccharide amylovoran, both of which are key compounds essential for biofilm formation in *E. amylovora*. Thus, ArcZ not only regulates motility through Lrp and FlhDC, but also influences biofilm formation in *E. amylovora* (Fig. 3; Schachterle and Sundin 2019).

**FIGURE 5.**
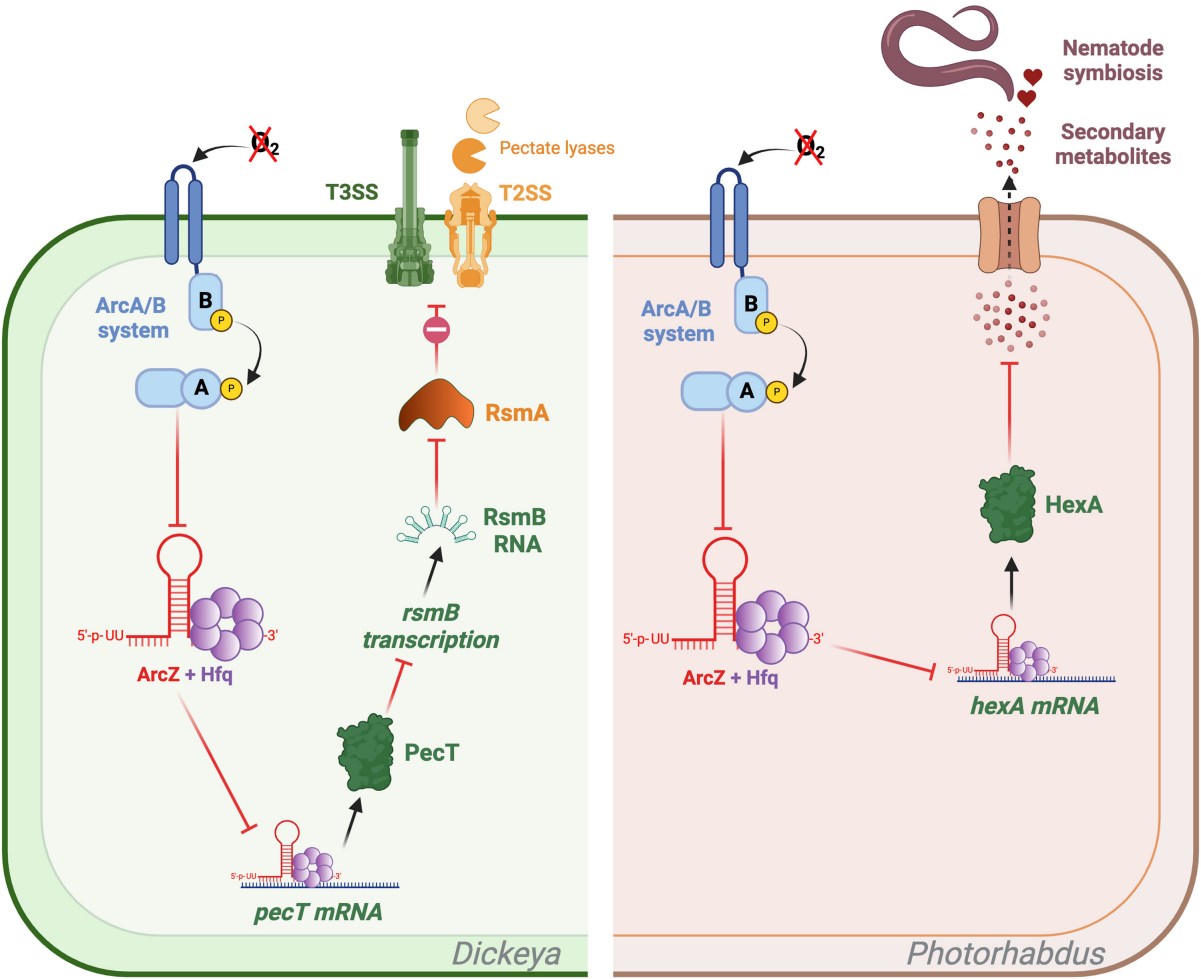
Regulation of *hexA*/*pecT* by ArcZ in *Dickeya* and *Photorhabdus*. Direct and indirect targets of ArcZ are shown in *Dickeya* (green panel) and *Photorhabdus* (brown panel). Green arrows indicate activation, red arrows indicate repression, and black arrows indicate either translation or activity. In *Dickeya*, ArcZ inhibits the translation of *pecT*, which prevents the inhibition of *rsmB* transcription by PecT. RsmB is a small Hfq-independent regulatory RNA that binds to RsmA and prevents it from repressing the expression of T3SS and pectinases. In *Photorhabdus*, ArcZ inhibits the translation of *hexA* and thus enhances the production of secondary metabolites, which are essential for nematode symbiosis. Figure was created with BioRender.com.

In the *Pectobacteriaceae* family of plant pathogenic bacteria, one of the key regulators of virulence factor synthesis is named PecT in *D. dadantii* (Condemine et al. 1999) or HexA in *P. carotovorum* (Mukherjee et al., 2000). PecT represses the expression of the sRNA RsmB, a Hfq-independent small regulatory RNA. The *rsmB* RNA binds to RsmA, an RNA binding protein, preventing it from repressing the expression of its target genes, including those encoding the main virulence factors, the T3SS and pectinases, which are plant cell wall-degrading enzymes (PCWDEs) (Hyytiäinen et al. 2001; Yang et al. 2008). It has been demonstrated that ArcZ directly represses the translation of *pecT*. This repression results in an increase in the amount of *rsmB* available to titrate RsmA, leading to increased production of the T3SS and of the PCWDE (Fig. 5; Yuan et al. 2019). Consequently, an *arcZ* mutant in *D. dadantii* has a drastically reduced virulence (Yuan et al. 2019). A mutant of *P. carotovorum* lacking *arcZ* was also found to be less virulent. However, the targets of ArcZ in this bacterium were not investigated (Wang et al. 2018).

Similarly, ArcZ induces the expression of the secondary metabolite clusters *sol* and *zms* in *Dickeya solani*, resulting in the production of the antimicrobial molecules solanimycin and zeamine, respectively. Nevertheless, the precise targets of ArcZ in *D. solani* are still unknown, although it is suspected that *pecT* may be involved, as its target site in the 5′ UTR of *pecT* is conserved (Brual et al. 2023).

Secondary metabolites play a significant role in the mutualistic associations of nematodes with *Xenorhabdus* and *Photorhabdus* (Tobias et al. 2017b). HexA, known for its ability to repress secondary metabolite production in *Photorhabdus* (Tobias et al. 2017a), is repressed by ArcZ. This is achieved through direct binding to *hexA* 5′-UTR mRNA in *Photorhabdus* and *Xenorhabdus*. Consequently, secondary metabolites that modulate the nematode immune response (Fig. 5) are produced more abundantly when ArcZ represses *hexA* translation (Fig. 5; Neubacher et al. 2020).

### ArcZ and the bacterial metabolism

A few genes targeted by ArcZ that play a role in nutrient metabolism or transport have been identified. In *E. coli*, the *ppsA* gene encodes a phosphoenolpyruvate synthetase that is required for the conversion of pyruvate to phosphopyruvate, which initiates the process of gluconeogenesis. ArcZ directly or indirectly regulates *ppsA* in a positive manner. Hence, ArcZ regulates gluconeogenesis initiation by activating *ppsA* transcription or translation when *E. coli* is grown in the presence of pyruvate (Rachwalski et al. 2022).

Another potential target involved in specific serine transport in *S. typhimurium* is *sdaC*, which is directly repressed by ArcZ at the mRNA level. In this case, *sdaC* is cotranscribed with *sdaB*, a serine deaminase, suggesting that SdaCB is involved in serine catabolism (Fig. 4). Thus, ArcZ may play a potential role in repressing serine catabolism (Papenfort et al. 2009).

## MOLECULAR MECHANISMS USED BY ArcZ TO REGULATE TRANSLATION

**FIGURE 6.**
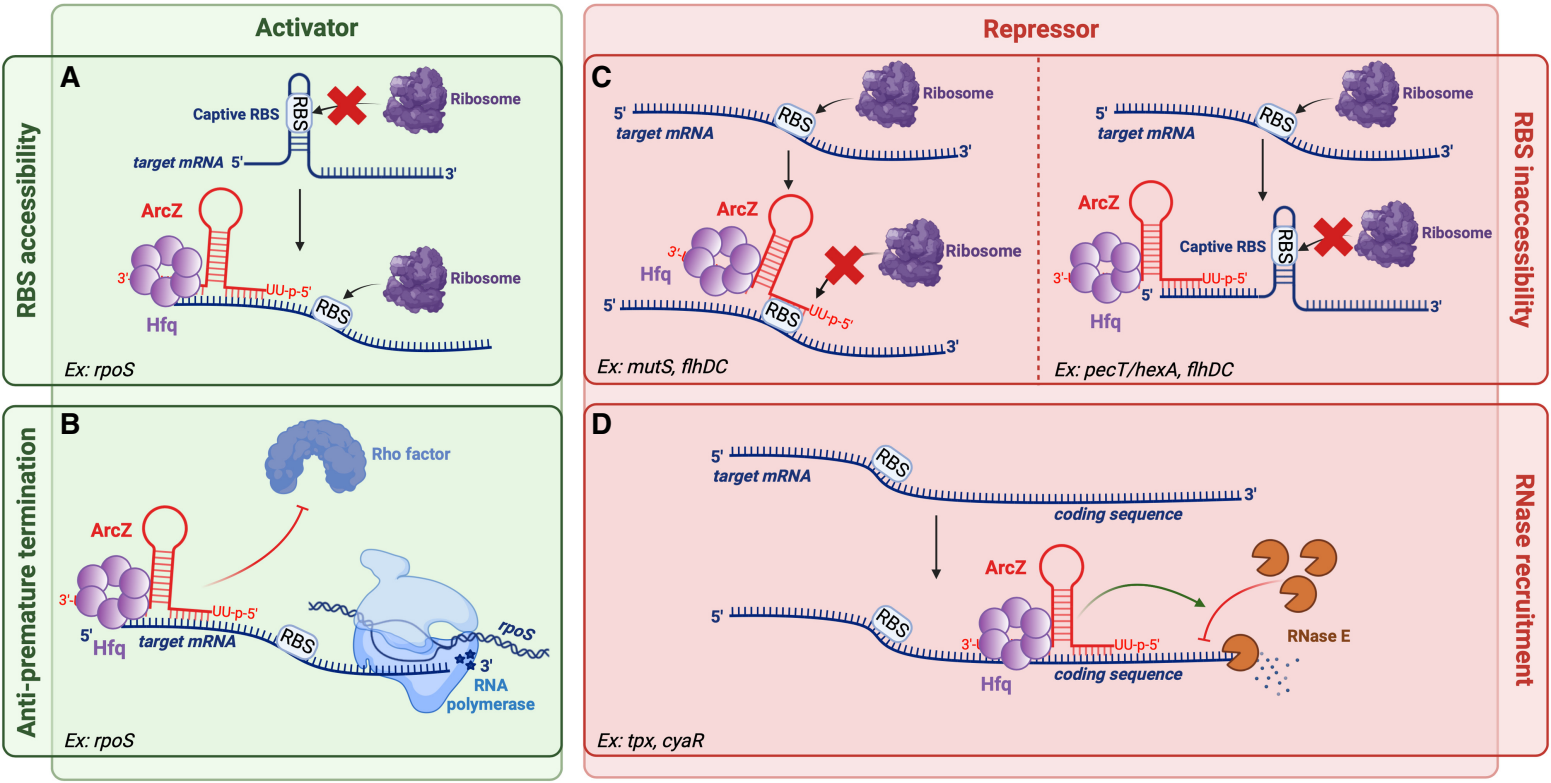
ArcZ's modes of action. ArcZ can activate the translation of mRNA (green panel). (*A*) ArcZ interacts with the 5′-UTR region of the *rpoS* mRNA and releases its ribosome-binding site (RBS), which is initially involved in a hairpin structure, allowing for the translation of *rpoS*. (*B*) Additionally, during the transcription of *rpoS*, ArcZ prevents the Rho termination factor from binding to the mRNA, thus blocking premature termination. ArcZ can also repress the translation of mRNA (red panel), such as with *mutS* or *flhDC*. (*C*) ArcZ can repress translation by binding to the RBS of the mRNA, preventing access of the ribosome. Additionally, it can bind upstream of the RBS, such as with *pecT* or *flhD*, forming a secondary structure that is incompatible with translation. (*D*) ArcZ can also bind to the coding region of the mRNA or sRNA, making it more susceptible to RNase such as with *tpx* and *cyaR*. Figure was created with BioRender.com.

As previously shown, ArcZ possesses the ability to inhibit or activate its target mRNAs. The processed 56 nt transcript that binds to mRNAs is highly conserved (Fig. 1A). This transcript is capable of regulating targets in diverse ways (Fig. 6). The various targets can be categorized into five groups based on the ArcZ pairing location in the target mRNA: (1) ArcZ can pair at the RBS or in close proximity to it, such as with the *mutS* mRNA; (2) it can pair in the coding region of a gene such as with the *tpx* mRNA; (3) it can pair in the 5′-UTR region of the mRNA ∼50 nt from the AUG, as demonstrated with *pecT* or *flhDC*; (4) it can prevent Rho-mediated premature termination; and (5) it can pair directly with an sRNA like CyaR.

### Binding of ArcZ near the RBS

In the case of *mutS*, ArcZ pairs near to the RBS to repress the translation of *mutS*. In fact, after being made mature and stable by Hfq and RNase E, ArcZ will pair at the level of the 5′-UTR of *mutS* between positions −25 and −15 relative to the ATG, upstream of the RBS of *mutS* that is located from −11 to −5. This pairing is proposed to alter the secondary structure of the *mutS* translation initiation region, making it unavailable to ribosomes (Fig. 6; Chen and Gottesman 2017).

ArcZ can bind directly to the RBS region of STM3216 mRNA between position −25 and −5. A similar phenomenon is observed with the *sdaC* mRNA, where ArcZ hybridizes directly with the RBS of the region from −13 to −2. This type of pairing effectively masks the RBS, rendering it inaccessible for translation initiation (Papenfort et al. 2009).

### Binding of ArcZ in the coding region of the target mRNA

ArcZ can bind to the coding region of the *tpx* gene mRNA, specifically between positions +10 and +26. Inhibition may occur through the inhibition of 30S ribosome subunit association, as well as through the degradation of the mRNA via RNase recruitment (Fig. 6; Papenfort et al. 2009). A similar mechanism is observed with the binding of the MicC sRNA to the coding region of the *ompD* mRNA, resulting in the repression of *ompD* translation due to accelerated degradation by RNase E (Pfeiffer et al. 2009).

### Binding of ArcZ upstream of the RBS

In *Photorhabdus*, ArcZ binds to a 9 nt region located on the 5′ UTR of the *hexA* mRNA between positions −50 and −42 (Neubacher et al. 2020). This results in the inhibition of *hexA*’s translation. The region of binding is distal to the RBS, making it improbable for ArcZ to prevent the RBS's recognition in a way comparable to that observed for *mutS*. Hence, the inhibition mechanism of ArcZ is different from that of *mutS*. The regions where ArcZ pairs in the 5′ UTR of *hexA* are rich in C and A nucleotide bases. This type of sequence in the 5′ UTRs of mRNAs can enhance translation (Sharma et al. 2007). Previous research suggests that the GcvB sRNA can regulate several ABC transporter mRNAs using this mechanism (Sharma et al. 2007). The hypothesis is that ArcZ, by binding to these C/A-rich regions, covers up these translation enhancers and thus decreases the translation of *hexA* mRNA (Neubacher et al. 2020).

To repress *pecT* in *D. dadantii*, ArcZ pairs at a region located between positions −84 and −58 from the start codon, which is considerably far from the RBS. Additionally, the region involved in the pairing is not rich in C/A. Therefore, this repression could be due to a modification of the secondary structure of *pecT* mRNA leading to the formation of a hairpin structure that masks ribosome access to the RBS (Fig. 6; Yuan et al. 2019).

### Dual site binding of ArcZ in the 5′-UTR *flhDC* mRNA in *E. coli*

In *E. coli*, the interaction of ArcZ with the 5′-UTR region of *flhDC* is more complex than the previously cited cases. Actually, the same region of ArcZ pairs with two sites in the 5′ UTR of *flhDC*: one located between nucleotides −23 and −7, in close proximity to the RBS of *flhD*, and the other situated between positions −63 and −47. The farthest region from the RBS corresponds to a region exhibiting high C/A levels. A hypothesis proposes that ArcZ, by pairing, removes a hairpin structure predicted for the *flhDC* mRNA in this region, changing the steric constraints for base stabilization at the RBS level (Fig. 6). Nevertheless, it appears that the pairing of ArcZ at the location farthest from the RBS is the most potent in suppressing *flhDC* translation (De Lay and Gottesman 2012; Schachterle et al. 2019b). It is noteworthy that the sRNA McaS activates the translation of *flhD* (Thomason et al. 2012), an effect that is opposite to that of ArcZ. However, McaS and ArcZ share a common binding site between positions −61 and −52 on the *flhD* 5′ UTR (De Lay and Gottesman 2012). McaS, like ArcZ, has a second binding site on the 5′ UTR of *flhD*, but further upstream of the start codon between positions −86 and −77 on the *flhD* 5′ UTR. It was proposed that McaS may initially bind to an overlapping ArcZ site on *flhDC* mRNA, aiding a second McaS site to pair and expose the mRNA for ribosome entry (De Lay and Gottesman 2012; Thomason et al. 2012). This suggests that despite targeting the same area, two sRNAs can regulate differently the same target. Ultimately, ArcZ and McaS likely compete for the same *flhDC* mRNA site, affecting cell motility based on which sRNA prevails (De Lay and Gottesman 2012).

### ArcZ prevents Rho-mediated premature termination and liberates the RBS for *rpoS* translation

ArcZ linked to Hfq binds to the *rpoS* mRNA at the region −91 to −63 relative to the translation start. However, only 23 of the 28 bases in ArcZ match those in *rpoS* mRNA, so this binding is not perfect. In the absence of ArcZ, the RBS of *rpoS* is locked in a hairpin loop, making the RBS inaccessible to the ribosome and preventing translation initiation (Mandin and Gottesman 2010). By pairing in this region of the 5′ UTR of *rpoS*, ArcZ unfolds the hairpin loop and frees up the RBS, allowing for translation initiation (Fig. 6). Additionally, binding of DsrA, RprA, and ArcZ appears to stabilize *rpoS* mRNA in *E. coli* and protects it from degradation by RNase E (McCullen et al. 2010). In the absence of ArcZ, the Rho factor causes premature termination of *rpoS* transcription by binding to the 5′ UTR of *rpoS*. When ArcZ binds to the 5′ UTR of *rpoS* mRNA, it prevents interaction between the Rho factor and this region. Therefore, ArcZ functions as an antiterminator for transcription and thereby activates the translation of *rpoS* (Fig. 6; Sedlyarova et al. 2016).

### Binding of ArcZ directly to another sRNA

In *E. coli*, CyaR is a CRP-regulated RNA whose pairing to a region close to the ArcZ binding site in the 5′ UTR of *rpoS* mRNA leads to its degradation (Kim and Lee 2020). RIL-seq data revealed the presence of ArcZ–CyaR interactions (Melamed et al. 2016). The direct interaction between CyaR and ArcZ results in the degradation of CyaR by the RNase E, thereby alleviating the CyaR-mediated repression of *rpoS* and maximizing the activation of *rpoS* by ArcZ (Kim and Lee 2020).

## PERSPECTIVES

ArcZ is a major regulator of gene expression in *Enterobacterales*, as it regulates 10%–15% of the genome in various species. The Ril-seq technique, which allows the identification of sRNA–mRNA target pairs pulled down with the Hfq protein, has been used to identify more than 300 putative new ArcZ mRNA targets in *E. coli* and *S. enterica* (Melamed et al. 2016; Liu et al. 2023). Of note, 330 of the 335 base-pairing interactions identified involve the seed domain at the 5′ end of the processed form of ArcZ (Fig. 1A; Melamed et al. 2016). Known ArcZ targets were identified (*rpoS*, *flhD*, *sdaC*, and *tpx*). Although further validation is required for the majority of these targets, these studies provide compelling evidence that ArcZ plays a central role in regulating a wide variety of targets.

Determining ArcZ's targets in a new bacterial model remains challenging in silico. Prediction software of sRNA–mRNA interaction such as CopraRNA or IntaRNA (Wright et al. 2014) may fail to identify complex interaction targets, such as the interaction zones between ArcZ and *flhD*. Therefore, there are still numerous targets and regulatory mechanisms that are yet to be uncovered. Additionally, an interesting hypothesis has been proposed by Papenfort et al. (2009). These authors observed that ArcZ is an sRNA conserved in *Enterobacterales* and that its inhibitory or activating action on mRNA translation is in many cases achieved by pairing at the site of the SD sequence, the most conserved element of bacterial mRNAs. They suggested that when ArcZ is in high intracellular concentration and once ArcZ has paired with all of its primary targets, excess ArcZ molecules will be able to pair with secondary targets at the RBS. This type of regulation has been observed in the posttranscriptional regulatory machinery at the mammalian level (Saxena et al. 2003; Papenfort et al. 2009). If confirmed, this hypothesis will likely expand the range of ArcZ-controlled genes beyond expectation.

**FIGURE 7.**
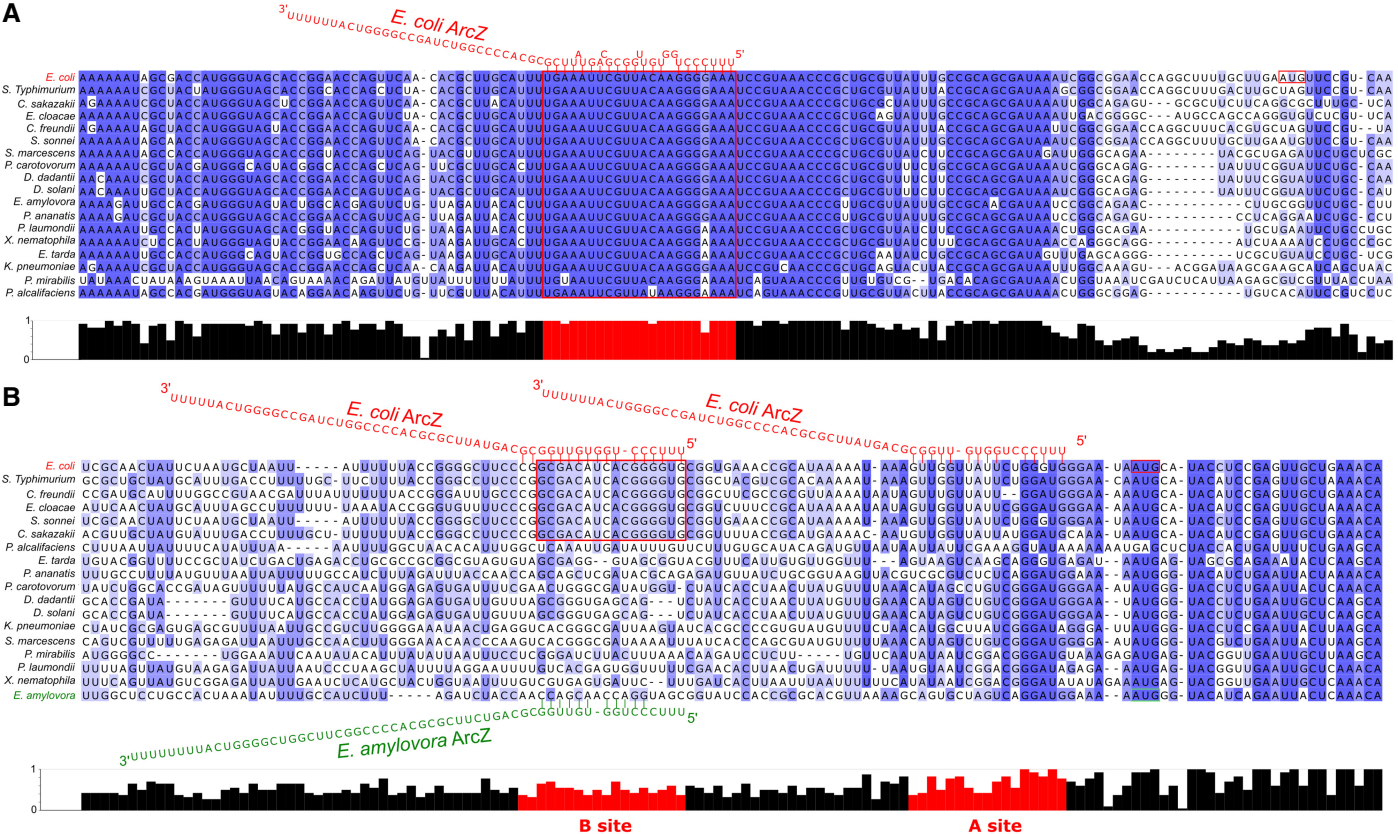
*rpoS* and *flhD*, two ArcZ targets conserved in *Enterobacterales*. Alignment of (*A*) *rpoS* mRNA sequence and (*B*) *flhD* mRNA sequence from *Pantoeae ananatis*, *Escherichia coli*, *Salmonella typhimurium*, *Klebsiella pneumoniae*, *Photorhabdus laumondii*, *Xenorhabdus nematophila*, *Dickeya dadantii*, *Pectobacterium carotovorum*, *Serratia marcescens*, *Proteus mirabilis*, *Erwinia amylovora*, *Providencia alcalifaciens*, *Edwardsiella tarda*, *Shigella sonnei*, *Citrobacter freundii*, *Enterobacter cloacae*, and *Cronobacter sakazakii* (genomes are the same as those used in Fig. 1). Conservation score is plotted *below*, and on this plot the interaction zone between ArcZ and *rpoS*/ArcZ and *flhD* shown in *E. coli* and *E. amylovora* is colored red. The interaction of *E. coli* ArcZ (in red) with *rpoS* and *flhD* mRNA and of *E. amylovora* ArcZ (in green) with *flhD* mRNA is shown (Mandin and Gottesman 2010; De Lay and Gottesman 2012; Schachterle et al. 2019b). The red squares correspond to ArcZ binding site regions conserved between *E. coli* and other bacteria. Alignments were performed using MUSCLE (Edgar 2004) and processed with Jalview (Clamp et al. 2004).

A remarkable observation concerning ArcZ is the remarkable degree of conservation of the seed region located after the cleavage site and, in mirror image, the high degree of conservation of the pairing region with the 5′ UTR of *rpoS*. Indeed, an alignment of the *rpoS* 5′ UTR in *Enterobacterales* (Fig. 7A) showed that the region of the 5′ UTR that pairs with ArcZ is highly conserved. Given that RpoS is a major regulator of the general stress response, it can be assumed that regulation of the stress response by ArcZ via RpoS is also conserved in most *Enterobacterales*. However, some nucleotide changes, of the order of one to two bases, exist in the seed region of ArcZ (Fig. 1A) as in *D. dadantii*, *P. carotovorum*, *E. amylovora*, and *E. tarda*. It can be observed that compensatory nucleotide changes for these mutations are not systematically found in the 5′ UTR of *rpoS* (Fig. 7A). This suggests that the ArcZ–*rpoS* pairing may be less stable in these bacteria. It would be of interest in the future to conduct a systematic analysis to verify the interactions between ArcZ and *rpoS* 5′ UTR in bacteria containing nucleotide modifications in either *arcZ* or *rpoS*. In some cases, the interaction may no longer occur. In addition, the conservation of pairing between the seed region of ArcZ and other targets is less clear. For instance, an alignment of the 5′ UTR of *flhD* in different *Enterobacterales* species (Fig. 7B) revealed that this pairing is less conserved than the ArcZ/*rpoS* pairing. It is therefore possible that ArcZ does not regulate motility via the control of *flhD* in some of these bacteria, or does not regulate motility at all. Nevertheless, in *E. amylovora*, although the 5′ UTR of *flhD* is different from that of *E. coli*, an interaction could be detected (Fig. 7B; Schachterle et al. 2019b). This putative pairing zone is not conserved in *D. dadantii*, whereas ArcZ deletion results in a difference in motility of the mutant compared to a wild-type strain. It was proposed that regulation of motility in *D. dadantii* by ArcZ occurs through an as yet unknown mechanism (Yuan et al. 2019). A significant amount of work remains to be done before a clear picture can be drawn of the manner in which ArcZ fulfills its regulatory role in each of the major bacterial genera of *Enterobacterales*.

**FIGURE 8.**
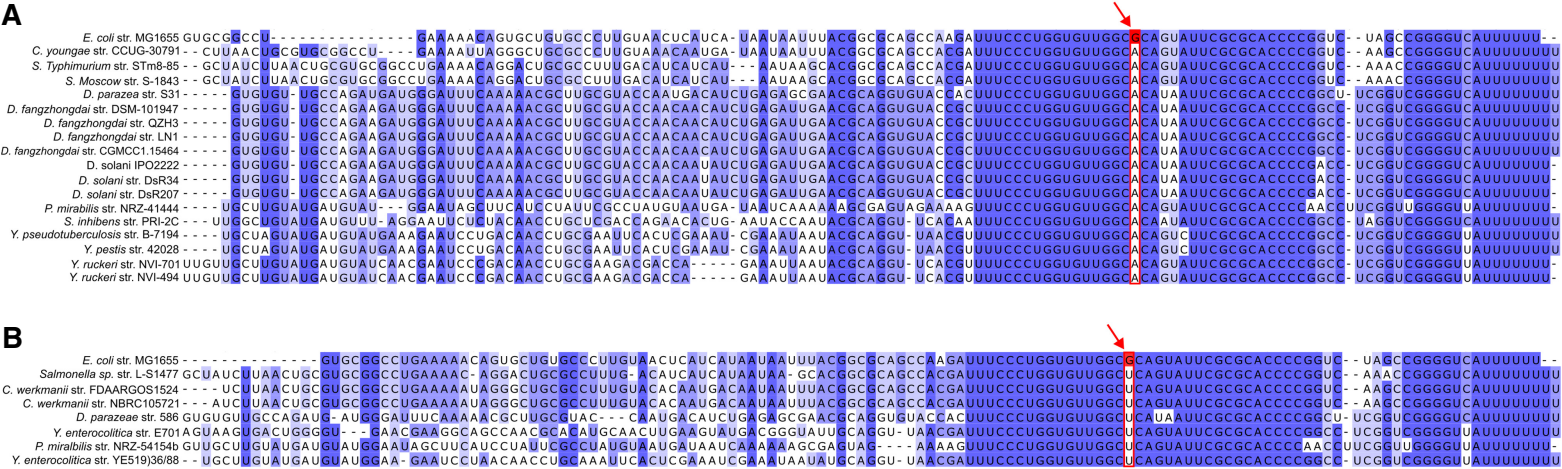
Alignments of mutated *arcZ* alleles in *Enterobacterales*. A BLASTN analysis was conducted on the *enterobacterales* NCBI RefSeq genomes using the conserved nucleotide sequence that contains the mutation (*A*) G to A or (*B*) G to U at position 90, in comparison with the *Escherichia coli* MG1655 reference genome. The mutation is indicated by a red arrow and surrounded by a red rectangle. Alignments were performed using MUSCLE (Edgar 2004) and processed with Jalview (Clamp et al. 2004).

A single-point mutation was found in the 3′ region of ArcZ in the *D. solani* type strain IPO2222 (Brual et al. 2023). This G-to-A mutation is positioned 17 bases downstream from the RNase E cleavage site. Despite being distant from the cleavage site, it prevents processing of the full-length ArcZ_IPO2222_ into a short, stable form. Consequently, ArcZ_IPO222_ is not functional. The predicted secondary structure of the full-size form of ArcZ_IPO222_ is altered in comparison with nonmutated ArcZ from other *D. solani* strains where ArcZ is active. This indicates the potential of some tertiary structures in small RNAs to prevent cleavage by RNase E. This is supported by the observation that when point mutations are introduced experimentally in ArcZ and alter its secondary structure, ArcZ variants may not be processed by the RNase E (Yuan et al. 2019). Notably, the ArcZ loss-of-function mutation found in *D. solani* IPO2222 is shared by other *Dickeya* species, including *D. fangzhongdai* and *D. parazeae* (Brual et al. 2023). Expanding the search for this mutation to other species reveals its presence in strains of *Citrobacter youngae*, *S. typhimurium*, *S. enterica*, *Proteus mirabilis*, *Yersinia pestis*, *Y. pseudotuberculosis*, and *Y. ruckeri*. Another G-to-T mutation has been found at the same location in other strains (Fig. 8). It would be worthwhile to compile a comprehensive list of *arcZ* alleles in *Enterobacterales* and investigate their systematic processing by the RNase E.

Why do certain isolated strains exhibit *arcZ* mutations that result in functional loss? It cannot be ruled out at present that the isolation of these bacteria on a nutrient-rich laboratory medium has resulted in the selection of a mutated strain in *arcZ*. Bacteria cultivated in laboratory conditions are known to accumulate mutations (Nahku et al. 2011; Spira et al. 2011; Chandler et al. 2019; Artuso et al. 2022; Jacques et al. 2023), some of these mutations, particularly those in *arcA* and *rpoS*, which are two prime targets of ArcZ, contribute to enhancing the catabolism of amino acids that are abundant in rich environments (Saxer et al. 2014). Nevertheless, mutations in *arcZ* have not been isolated during experimental evolution assays in vitro. Another possible hypothesis is that mutations in *arcZ* can lead to the emergence of cheaters. Cheaters are individuals who do not cooperate with others in the population, but still benefit from the public goods generated by cooperators without contributing to the costs of producing those goods (Smith and Schuster 2019). Since ArcZ regulates a variety of genes associated with flagellum apparatus and secretion systems machineries in various bacteria, it is plausible that bacterial cells with *arcZ* defect can benefit from wild-type cooperators’ secretion in a host organism. The secretion of virulence factors can be costly for bacteria. For example, *S. typhimurium* mutants deficient in T3SS cannot cause independent mouse infections, but they outcompete isogenic wild-type bacteria during coinfections (Diard et al. 2013). In co-infection experiments within a host, it would be interesting to investigate whether *arcZ*-deficient mutants could outcompete the wild-type strain.
